# Remission in rheumatoid arthritis: benefit over low disease activity in patient-reported outcomes and costs

**DOI:** 10.1186/ar4491

**Published:** 2014-02-21

**Authors:** Helga Radner, Josef S Smolen, Daniel Aletaha

**Affiliations:** 1 Medical University Vienna, Department of Internal Medicine III, Division of Rheumatology, Waehringer Guertel 18-20, A-1090 Vienna, Austria

## Abstract

**Introduction:**

Rheumatoid arthritis (RA) is a chronic inflammatory disease that causes a considerable burden for the patient and society. It is not clear yet whether aiming for remission (REM) is worthwhile, especially when compared with low disease activity (LDA).

**Methods:**

In 356 consecutive RA patients, we obtained data on physical function (health assessment questionnaire (HAQ)), health-related quality of life (HRQoL: Short Form 36 (SF36), Short Form 6 dimensions (SF-6D), Euro QoL 5D (EQ-5D)), work productivity (work productivity and activity impairment questionnaire (WPAI)), as well as estimation of direct and indirect costs. Cross-sectionally, data were compared in patients within different levels of disease activity according to the simplified disease activity index (SDAI; remission (REM ≤3.3); *n* = 87; low disease activity (LDA: 3.3 < SDAI ≤11); *n* = 103; moderate to high disease activity (MDA/HDA) >11 *n* = 119) by using analyses of variance (ANOVA). Longitudinal investigations assessed patients who changed from LDA to REM and vice versa.

**Results:**

We found differences in patients achieving REM compared with LDA for HAQ (0.39 ± 0.58 versus 0.72 ± 68), WPAI (percentage impairment while working 11.8% ± 18.7% versus 26.8% ± 23.9%; percentage of overall activity impairment, 10.8% ± 14.1% versus 29.0% ± 23.6%)), EQ-5D (0.89 ± 0.12 versus 0.78 ± 0.6) and SF-36 (physical component score (PCS): 46.0 ± 8.6 versus 38.3 ± 10.5; mental component score (MCS): 49.9 ± 11.1 versus 47.9 ± 12.3) (*P* < 0.01 for all, except for SF36 MCS). Regarding costs, we found significant differences of direct and indirect costs (*P* < 0.05) within different levels of disease activity, with higher costs in patients with higher states of disease activity. Longitudinal evaluations confirmed the main analyses.

**Conclusion:**

Patients with REM show better function, HRQoL, and productivity, even when compared with another good state, such as LDA. Also from a cost perspective, REM appears superior to all other states.

## Introduction

Rheumatoid arthritis (RA) is a chronic, inflammatory, destructive joint disease. RA is thus associated with major consequences for the individual, causing loss of function and work disability, and poses significant challenges to society, given its economic consequences. This socioeconomic burden of RA has been the focus of many studies and reviews, particularly in the more recent decades [1,2]. Traditional and biologic disease-modifying antirheumatic therapies (DMARDs) may reduce this burden considerably [3-5].

The best clinical outcome of RA is the achievement of remission, a state of no or very little inflammatory disease activity. This state is linked to a total halt of progression of joint damage [6,7] as well as to a maximally possible reversal of chronic disability [8]. Because disability, as evaluated by measures like the health assessment questionnaire disability index (HAQ), is tightly associated with working capacity [9,10], improving physical function not only will benefit the patient but also will improve major socioeconomic consequences of RA.

Current therapeutic goals are aimed at achieving remission, but allow low disease activity as an alternative goal, recognizing that remission may not be reasonably achievable in most patients with RA, especially in established disease [11,12]. Both of these states provide significant benefit to patients who have gone through the anguish of high disease activity. It is still unclear, however, whether better outcomes of the disease may compensate for potentially higher direct medical costs, particularly if expensive biologic therapies are involved [13,14]. Moreover, it is unknown to what extent the presumed small step from low disease activity to remission is relevant regarding patient-reported outcomes and costs. It was therefore the purpose of the present study to investigate whether attaining a state of remission conveys a benefit when compared with low disease activity from a patient perspective, as well as from a socioeconomic point of view, by using a population of routine clinic patients.

## Methods

### Patient population

At our clinic, patients with RA are routinely seen about every 3 to 4 months, and their clinical and laboratory variables are documented in a longitudinal database [15]. Variables regularly documented include CRP, ESR, numbers of swollen and tender joints by using a 28-joint count (SJC28, TJC28); patient global assessment of disease activity (PGA), evaluator global assessment of disease activity (EGA), pain by visual analogue scale, and physical function by HAQ. Composite indices, such as the simplified and clinical disease activity indices (SDAI, CDAI) and the Disease Activity Score using 28-joint counts (DAS28) were calculated based on these variables, according to the following formulae:

CDAI = SJC28 + TJC28 + PGA(in cm) + EGA(in cm) [16]

SDAI = SJC28 + TJC28 + PGA(in cm) + EGA(in cm) + CRP(mg/dl) [17]

$$\mathrm{DAS}28=0.56*\sqrt{\left(\mathrm{TJC}28\right)}+0.28*\sqrt{\left(\mathrm{SJC}28\right)}+\mathrm{In}\left(\mathrm{ESR}\right)+0.014*\mathrm{PGA}$$

[18]

Cut points used to separate the states of remission, and low, moderate, and high disease activity are as follows: 2.8, 10, and 22 for CDAI; 3.3, 11, and 26 for SDAI; and 2.6, 3.2, and 5.1 for DAS28 [19].

Between May 2008 and March 2009, 356 consecutive consenting RA patients were included in our study. Patients were eligible if diagnosed as having RA by a rheumatologist, and no further inclusion or exclusion criteria were set forth to study a broad spectrum of typical RA patients, reflecting daily life. In addition to routinely performed assessments of disease activity, information regarding health-related quality of life (HRQoL), fatigue, and work productivity was obtained at consecutive visits. Furthermore, data on resource utilization and treatment costs were evaluated by a self-administered questionnaire to calculate direct costs for each patient. Indirect costs were deduced from the HAQ values, as described by Huscher *et al*. [20].

### Outcome measures

#### Health-related quality of life

First, we used the Short Form 36 (SF-36) [21,22], a questionnaire comprising 36 items, organized into eight domains: physical function (PF), physical role (RP), bodily pain (BP), general health perception (GHP), vitality (VT), social function (SF), emotional role (RE), and mental health (MH); these domains can be further aggregated into two summary measures, the physical component score (PCS; including PF, RP, BP, and GHP) and the mental component score (MCS; VT, SF, RE, and MH). SF-36 results are normalized, and lower levels represent more impairment and thus worse outcome.

Second, Short Form 6D (SF-6D) [23] was assessed, which is a revised form comprising six domains (SF, PF, RE, BP, MH, and VT) and a total of 11 items of the SF-36. For our analyses, we used the standard (4-week recall version) German Version 1.0. The six dimensions each have between two and six levels. An SF-6D “health state” is defined by selecting one level from each dimension; a total of 18,000 possible health states can thus be defined. The SF-6D preference-based measure can be regarded as a continuous outcome scored on a 0.29 to 1.00 scale, with 1.00 indicating full health.

Finally, we applied the Euro-QoL 5D (EQ-5D [24]), which constitutes a preference-based measure comprising five domains with three levels ranging from −0.53 to 1.00, with 1.00 indicating perfect health.

#### Work productivity

We used the Work Productivity and Activity Impairment Questionnaire (WPAI) consisting of six questions, to assess absenteeism and presence at paid work as well as in unpaid activity. We used the German-Austrian version of the WPAI-RA v2.0. WPAI outcomes are expressed as impairment percentages, with higher numbers indicating greater impairment and less productivity (that is, worse outcomes) [25].

#### Estimation of direct costs

To evaluate direct costs, resource use was recorded by using a questionnaire capturing the following items: inpatient stays at hospital and rehabilitation centers, surgery, imaging, and doctors’ visits. For cost of medication, only synthetic and biologic DMARDs were taken into account by using the reference costs as provided by the major healthcare provider for the Vienna area (Wiener Gebietskrankenkasse); other medical treatments (such as nonsteroidal antirheumatic drugs, glucocorticoids, and others) were not considered. Nondrug treatments like physical therapy were also evaluated. Furthermore, we collected data on costs of home adaptations, transportation, and home help that had been incurred by the patients. The items collected were based on a cost-effectiveness article and a systematic literature review on cost in RA [26,27] and can be found in the supplement [see Additional file 1]. All items were recorded for the 3-month period preceding the index visit and multiplied by 4 to estimate annual costs.

#### Estimation of indirect costs

Indirect costs were assessed by using the data of Huscher *et al*. [20] on mean annual costs of sick leave within different levels of HAQ (HAQ ≤1.2, €856; 1.2 < HAQ ≤ 1.7, €3,212; HAQ > 1.7, €7,619); costs of work disability by using the human capital approach (HCA) [28] and friction cost approach (FCA) [29,30] were also estimated (HCA, HAQ ≤1.2, €4731; 1.2 < HAQ ≤ 1.7. €12,707; HAQ >1.7, €18,894; FCA, HAQ ≤1.2, €752; 1.2 < HAQ ≤ 1.7, €2,019; HAQ > 1.7, €3,002). The FCA in contrast to the HCA takes into account that no economy achieves full employment and that productivity losses are counted only until a person previously unemployed replaces the productivity of persons who lost their work. In FCA, this time period is set to 58 days [29,30].

### Statistical analyses

For cross-sectional analyses, we divided patients according to their levels of disease activity by SDAI into remission (REM SDAI ≤ 3.3; low disease activity (LDA) 3.3 < SDAI ≤ 11; moderate disease activity (MDA) 11 < SDAI ≤ 26; and high disease activity (HDA) SDAI > 26). We assessed the univariate relation between outcomes of HRQoL, productivity, fatigue, functional disability, and disease activity by Spearman correlation. With analyses of variance (ANOVAs), we investigated whether these outcomes were significantly different at different levels of disease activity defined by SDAI. Sensitivity analyses were performed by using CDAI and DAS28 as alternative composite measures of disease activity. To account for potential confounders, we extended ANOVA and performed a General Linear Model (GLM), including disease duration, as a covariate in the model. With a GLM, we were able to calculate estimated marginal means (EMMs), which depict the mean for each level of disease activity, adjusted for any variable used in the model. For sensitivity analyses, we divided our patients into two groups: early RA, disease duration ≤2 years; late RA, disease duration >2 years; and rerun ANOVA within those two groups. Differences in costs between different levels of disease activity were compared by using a Kruskal-Wallis test.

In a final longitudinal analysis, we investigated whether patient-reported outcomes differed significantly in those patients who improved from LDA to REM, or who worsened from REM to LDA, by using a Student *t* test.

The patients involved consented to take part in the study, and the study was approved by the Ethics Committee of the Medical University Vienna. The Statistical Package for the Social Sciences (SPSS, Version 19) was used to conduct the analyses.

## Results

In total, 716 visits of 356 patients were documented; the median was two visits per patient (range, one to four); 209 patients had at least one follow-up visit within the time frame of this study (that is, 1.4 years). Patients’ characteristics at the baseline visit are depicted in Table 1.

**Table 1:** Patient characteristics at the baseline visit

**Patients *n*** **= 356**	
Female (%)	79.8%
Age (years)	59.9 ± 12.7
Disease duration (years)	11.5 ± 10.2
Rheumatoid factor positive(%)	59%
C-reactive protein (mg/dl)	2.1
Erythrocyte sedimentation rate (mm/hour)	25.9 ± 21.9
Swollen-joint count 28	2.1 ± 3.0
Tender-joint count 28	2.1 ± 3.8
Visual analogue scale pain (mm)	28.0 ± 22.4
Patient global assessment of disease activity (mm)	30.0 ± 23.3
Evaluator global assessment of disease activity (mm)	11.0 ± 13.7
Clinical disease activity index	8.3 ± 7.3
Simplified disease activity index	9.1 ± 7.7
Disease activity score 28	3.2 ± 1.2
Health-assessment questionnaire	0.81 ± 0.76
Euro QolL5D	0.77 ± 0.19
Physical component score SF-36	37.6 ± 11.3
Mental component score SF-36	48.0 ± 12.0
Patients currently employed (%)	26.5%
Activity impairment due to problem (%)	35.9 ± 26.7
Work time missed due to RA (%)	7.7 ± 24.5
Impairment while working due to problem (%)	27.8 ± 26.8
Overall work impairment due to RA (%)	2.7 ± 10.5

Values reported as means ± standard deviation, unless indicated otherwise.

### Measures of function, health-related quality of life, and productivity correlate significantly with disease-activity levels

Patient-reported outcomes of functional disability, HRQoL, productivity, and fatigue correlated significantly with disease activity according to SDAI, CDAI, and DAS28. The highest correlati*o*n was found between disease-activity scores and outcomes of functional disability (HAQ *r* = 0.54, 0.53, 0.53; SF-36 PCS *r* = −0.58, -0.56, -0.55; for SDAI, CDAI and DAS28 respectively, *P* < 0.01), as well as the ability to perform regular daily activity (percentage activity impairment, *r* = 0.52, 0.54, 0.56; for SDAI, CDAI, and DAS28 respectively, *P* < 0.01), whereas only a low correlation was seen between disease-activity scores and mental function (SF-36 MCS *r* = −0.15, -0.19, and -0.20, respectively, *P* < 0.01). Furthermore, HRQoL and fatigue correlated significantly with disease-activity measures (SF-6D r = −0.46, -0.47, -0.47; EQ-5D r = −0.51, -0.51, -0.48; VAS fatigue r = 0.45, 0.44, 0.45, respectively, p < 0.01). In currently employed patients, a good correlation was observed between “percent impairment while working due to RA” and disease activity (r = 0.51, 0.54, 0.56; for SDAI, CDAI and DAS28, respectively; p < 0.01), “percent overall work impairment due to RA” (r = 0.53, 0.53, 0.54, p < 0.01), and “percent work time missed due to RA” (r = 0.34, 0.34, 0.24, respectively, p < 0.01).

### Remission is associated with significantly better physical function, and health related quality of life than higher disease activity states including low disease activity

Looking at states of disease activity according to SDAI at the baseline visit, 24.4% (n = 87) were in REM, 42.1% (n = 150) in LDA, 28.9% (n = 103) in MDA and 4.5% (n = 16) in HDA. Given the small number of patients in HDA, we combined patients with MDA and HDA for further analyses.

**Figure 1:**
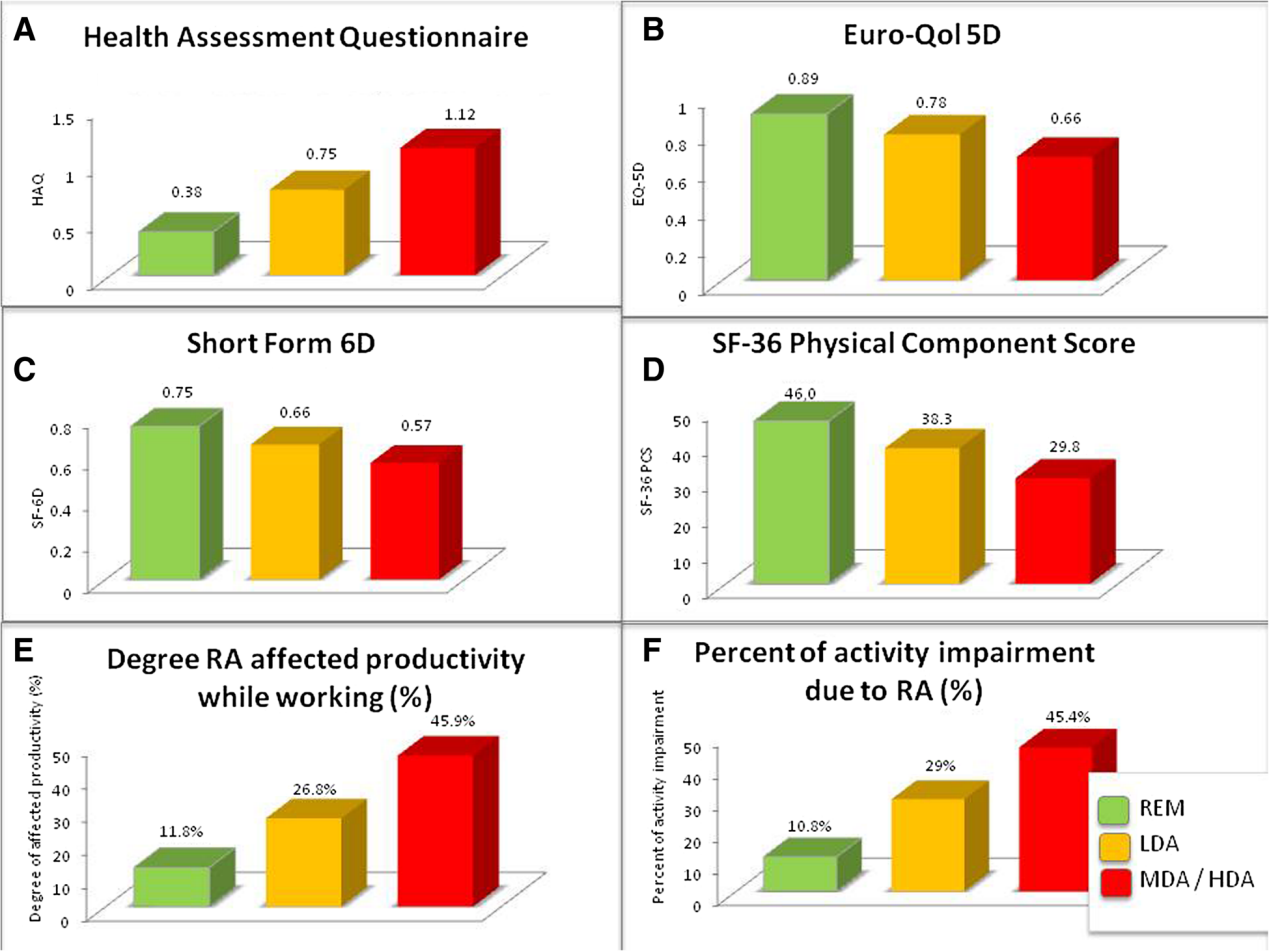
**Analyses of variance.** Panels depict significant differences of mean scores of Short-Form 6D **(A)**, Euro-QoL 5D **(B)**, Health Assessment Questionnaire **(C)**, Short-Form 36 Physical Component Score **(D)**, mean percentage of degree RA affects you while working **(E)**, and mean percentage of activity impairment while working **(F)** within levels of disease activity determined by SDAI (remission REM ≤ 3.3; 3.31 < low disease activity (LDA) ≤ 11; 11.01 < moderate to high disease activity (MDA/HDA)).

**Figure 2:**
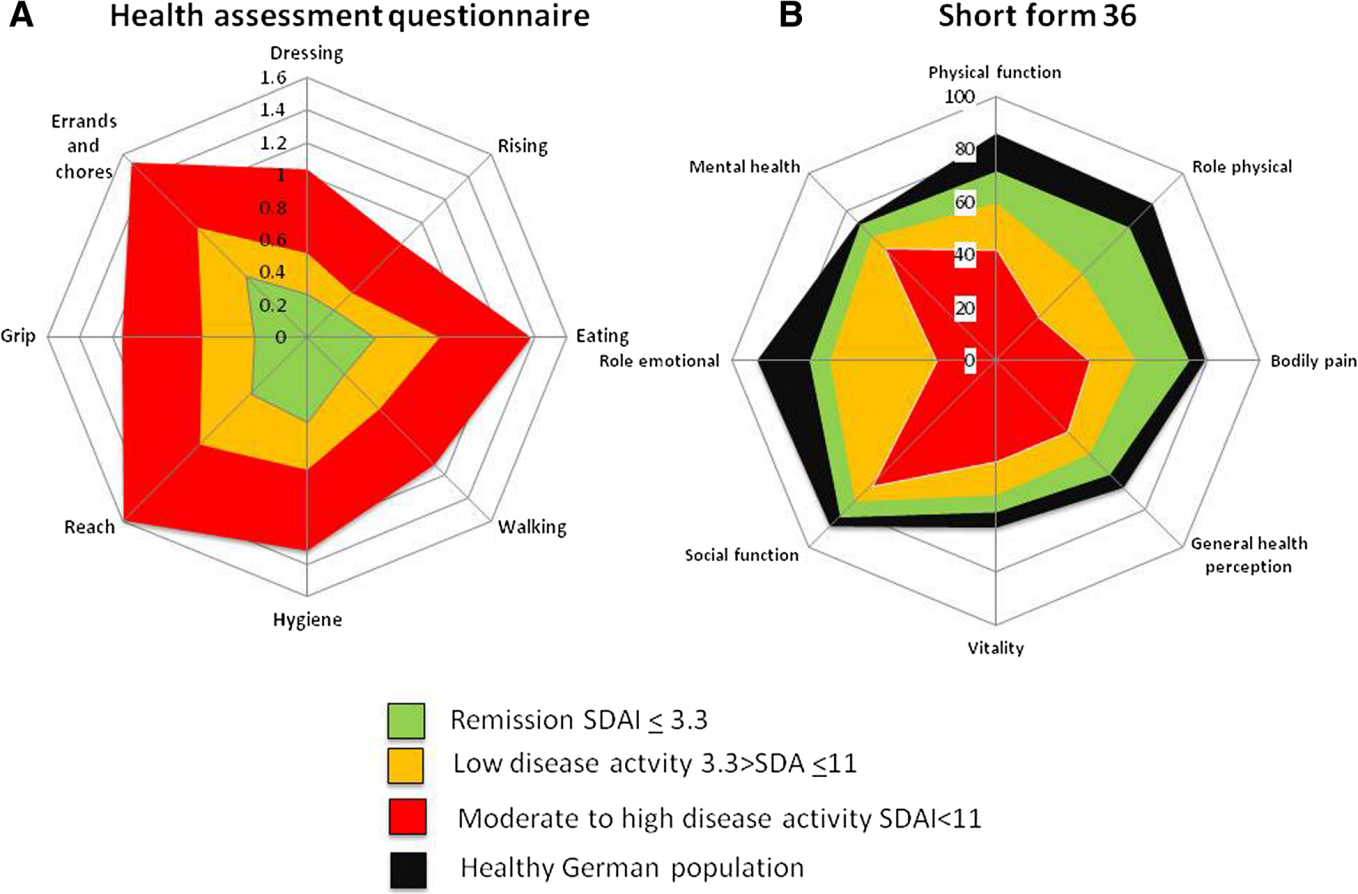
**Analyses of variance: Significant differences of different domains of Health Assessment Questionnaire (HAQ, A) and SF-36 physical component Score (PCS, score of 0 indicates poorest status, and 100 indicates best status; B) among levels of disease activity determined by SDAI (remission REM < 3.3; 3.31 < low disease activity (LDA) < 11; 11.01 < moderate to high disease activity (MDA/HDA)).**

Comparing functional disability by HAQ at the three levels of disease activity, we observed significant differences between REM, LDA and MDA/HDA (by SDAI), showing a HAQ increase with increasing disease activity. These results were not only obtained for the total HAQ-score (mean ± SD: REM 0.39 ± 0.58; LDA 0.72 ± 0.68; MDA/HDA 1.24 ± 0.75; (Figure 1A), but also for the different domains of the HAQ (Figure 2A).

Again, similar findings were obtained in a sensitivity analysis when assessing disease activity states by CDAI (mean ± SD HAQ: CDAI-REM 0.38 ± 0.56; LDA 0.75 ± 0.70; MDA/HDA 1.23 ± 0.74; p < 0.01) and DAS28 (HAQ mean ± SD: REM 0.46 ± 0.62; LDA 0.60 ± 0.66; MDA/HDA 1.24 ± 0.74; p < 0.01); however, the difference between HAQ-values in DAS28 REM and DAS28 LDA were not significant for the total HAQ score nor for the individual domains (data not shown), presumably because – in line with previous observations [31,32] - some patients in DAS28 REM have significant residual disease activity reducing the difference between the REM and LDA states by this score; indeed, HAQ and other values in DAS28 REM were numerically higher than those seen in the more stringent CDAI and SDAI remission criteria.

Assessing functional disability using the physical component score (PCS) of SF-36, we also found significant differences between the different levels of disease activity (SDAI-REM: 46.0 ± 8.6; LDA: 38.3 ± 10.5; MDA/HDA: 29.8 ± 9.2; p < 0.01). For the mental component score there were only subtle differences between disease activity states, but still a small trend toward better outcomes with lower disease activity (mental component score: SDAI-REM: 49.9 ± 11.1; LDA, 47.9 ± 12.3; MDA/HDA: 46.6 ± 12.3; *P* = 0.06). Significant differences were seen for all individual PCS domains of SF-36 between different levels of disease activity (Figure 2B).

When directly comparing LDA with REM, a significant difference was observed in the PCS of the SF-36 and in all domains except role emotional. Nevertheless, even when comparing RA patients in REM with a healthy German population, we did find significantly lower mean values for most domains (all except SF and MH; Figure 2B).

When we assessed potential differences in HRQoL by other measures, such as EQ-5D and SF-6D, we found very similar results, namely decreasing HRQoL with increasing levels of disease activity. For EQ-5D, the mean values ± SD were in SDAI-REM 0.89 ± 0.12, in LDA 0.78 ± 0.16 and in MDA/HDA 0.66 ± 0.21 (P < 0.001) (Figure 1B); for the SF-6D, the results were in REM, 0.75 ± 0.15, in LDA, 0.66 ± 0.14, and in MDA/HDA, 0.57 ± 0.12 (*P* < 0.001) (Figure 1C). Of note, the mean differences of patients in LDA by SDAI when compared with MDA/HDA were almost identical to the difference between REM and LDA.

In sensitivity analyses, we used CDAI and DAS28; in line with the previous results, these data showed significant differences between different levels of disease activity (CDAI SF-6D, mean ± SD: REM, 0.75 ± 0.15; LDA, 0.66 ± 0.13; MDA/had, 0.57 ± 0.12; CDAI EQ-5D, REM, 0.89 ± 0.12; LDA, 0.78 ± 0.16; MDA/had, 0.66 ± 0.21; DAS28 SF-6D: REM, 0.74 ± 0.15; LDA, 0.69 ± 0.14; MDA/had, 0.58 ± 0.12; DAS28 EQ-5D: REM, 0.88 ± 0.13; LDA, 0.81 ± 0.17; MDA/had, 0.67 ± 0.20; *P* < 0.01 for all analyses, respectively).

**Figure 3:**
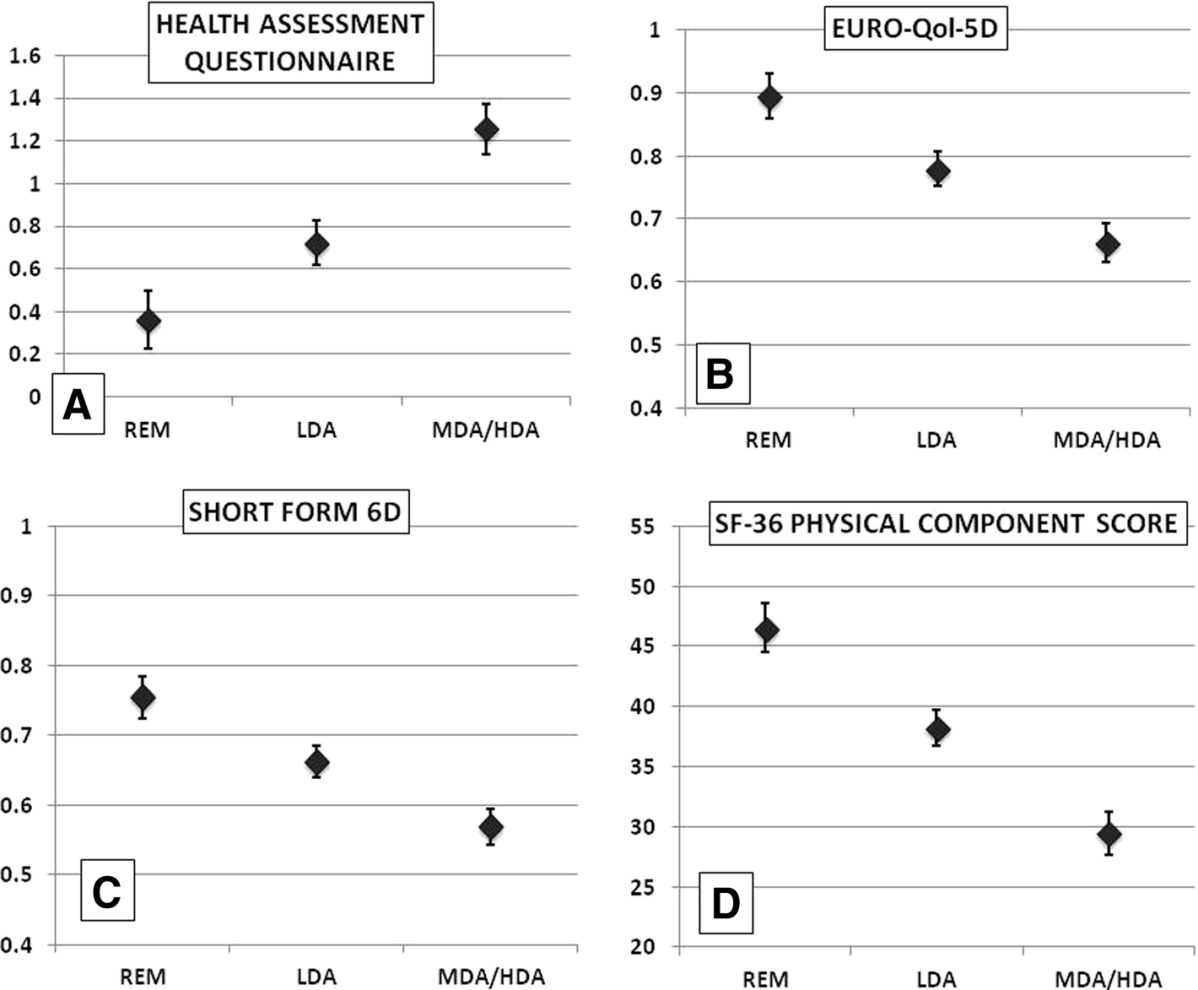
**General linear model adjusted for disease duration 11.34 years). (A)** Estimated marginal means (EMM) of Health Assessment Questionnaire for patients within remission (REM), low disease activity (LDA), and moderate to high disease activity (MDA/HDA) of an RA patient with 11.34 years of disease duration. **(B)** EMM of Euro-QoL 5D of respective patients. **(C)** EMM of Short Form 6D; **(D)** EMM of Short Form 36 physical component score. Each model showed significant differences (*P* < 0.01) of respective outcomes within patients of different levels of disease activity determined by simplified disease activity index (SDAI).

When extending ANOVA to a General Linear Model (GLM) that allows adjustment for disease duration, we again found significant differences (P < 0.01) of mean HAQ, SF-6D, EQ-5D, and PCS values between patients in REM, LDA, and MDA/HDA (estimated marginal mean (EMM) and 95% confidence interval are shown in Figure 3A through D).

In additional sensitivity analyses, we divided patients into two groups according to their disease duration: early RA (disease duration ≤2 years; *n* = 58) and established RA (disease duration >2 years, *n* = 298). Again, significant differences of HAQ, SF-6D, EQ-5D and PCS values were found between different levels of disease activity; in early as well as established RA patients (Table 2); notably, patients with established RA had worse levels in most measures compared with those with early RA.

**Table 2:** Significant differences of physical function and health-related quality of life between levels of disease activity (SDAI) in early (≤2 years) and late rheumatoid arthritis patients

	**HAQ**	**SF-6D**	**EQ-5D**	**SF-36 PCS**
EARLY RA				
REM (*n* = 7)	0.14 ± 0.2	0.74 ± 0.15	0.92 ± 0.10	42.7 ± 11.7
LDA (*n* = 26)	0.39 ± 0.42	0.70 ± 0.15	0.81 ± 0.16	41.8 ± 7.1
MDA/HDA (*n* = 25)	1.05 ± 0.81	0.60 ± 0.13	0.71 ± 0.19	33.1 ± 9.8
*P* Value	<0.01	0.01	0.01	0.006
LATE RA				
REM (*n* = 80)	0.41 ± 0.59	0.75 ± 0.15	0.89 ± 0.12	46.3 ± 8.3
LDA (*n* = 124)	0.78 ± 0.71	0.65 ± 0.14	0.77 ± 0.16	37.6 ± 10.9
MDA/HDA (n = 94)	1.30 ± 0.72	0.57 ± 0.11	0.65 ± 0.22	28.9 ± 8.8
*P* Value	<0.01	<0.01	<0.01	<0.01

*EQ-5D,* Euro Quality of life 5 dimensions; *HAQ,* Health Assessment Questionnaire; *LDA,* low disease activity; *MDA/HDA* moderate disease activity/high disease activity; *RA,* rheumatoid arthritis; *REM,* remission; *SF-6D,* Short Form 6 dimensions; *SF-36 PCS,* Short Form 36 Physical Component Score.

### Remission is associated with significantly higher levels of work productivity compared with low disease activity

In our cohort, 92 (26%) patients were currently employed; the majority were already retired (*n* = 209). Among the latter, 34% (*n* = 72) were in early retirement due to RA; 14 patients were unemployed; five of them specified that they were unemployed due to RA. Other work statuses reported: student (*n* = 3), maternity leave (*n* = 1), house wife/husband (*n* = 28); nine patients did not report anything.

Among currently employed patients, 30.4% were in SDAI REM (*n* = 28), 44.6% in LDA (*n* = 41) and 25% (*n* = 23) in MDA/HDA. With WPAI, we found significant differences in the “degree RA affected productivity while working” between levels of disease activity (%; mean ± SD): REM, 11.8% ± 18.7; LDA, 26.8% ± 23.9; MDA/HDA, 45.9% ± 28.6; *P* < 0.001; Figure 1E) and the percentage of activity impairment due to RA (mean + SD: REM, 10.8% + 14.1; LDA, 29.0 + 23.6; MDA/HDA, 45.4 + 25.0; *P* < 0.001; Figure 1F). No significant difference in absenteeism was found (percentage of work time missed because of RA) or the percentage of overall work impairment because of RA, although a numeric increase of work time missed and impairment was observed with increasing level of disease activity (data not shown).

### Costs

When comparing mean total direct costs between the three disease-activity states, we found significant differences (*P* = 0.05, ANOVA) and a similar trend regarding resource utilization (*P* = 0.06). Treatment costs were relatively similar across the different levels of disease activity (Table 3), with slightly higher costs in higher levels of SDAI. We also observed differences between REM, LDA, and MDA/HDA when comparing estimated indirect cost (Table 3) with significantly higher indirect costs due to sick leave and work disability in patients with higher levels of disease activity (*P* < 0.01).

**Table 3:** Mean annual costs (SD) in Euros within different levels of SDAI disease activity

	**Costs resource use**	**Treatment costs**	**Total direct costs**	**Costs sick leave**	**Costs work disability (HCA)**	**Costs work disability (FCA)**
**REM**	€828.28 (2,491.24)	€539.64 (729.46)	€1,367.92 (2,621.94)	€1,285.2 (1,502.1)	€5,772.8 (3,388.7)	€917.5 (538.3)
**LDA**	€1,039.02 (2,561.41)	€603.93 (762.06)	€1,642.95 (2,644.7)	€1,874.2 (2,185.9)	€7,186.6 (4,864.9)	€1,142.1 (772.8)
**MDA/HDA**	€1,702.39 (3,500.74)	€637.85 (748.46)	€2,340.24 (3,686.89)	€3,291.9 (2,871.3)	€10,525.7 (6,129.2)	€1,672.5 (973.7)
***P *** **value**	0.06	0.65	0.05	<0.01	<0.01	<0.01

*FCA,* friction cost approach; *HCA,* human capital approach; *HDA,* high disease activity; *LDA,* low disease activity; *MDA,* moderate disease activity; *REM,* remission.

Treatment costs include patients treated with biologic agents (overall, 36.2%. The distribution among different levels of disease activity was equal: REM, 32.2%; LDA, 36%; MDA/HDA, 39%).

### Moving from LDA to REM improves physical function and health-related quality of life in a longitudinal analysis

Within the period of observation (mean, 6.5 months), 23 patients changed their disease-activity states from LDA to REM. When investigating the changes in physical function, we saw significant improvement of HAQ from 0.61 ± 0.7 to 0.51 ± 0.7 (*P* = 0.005). Moreover, even though patients in LDA had already reached a relatively good outcome, fatigue improved from a score of 3.7 ± 2.1 to 2.9 ± 2.1 (*P* = 0.037). When we looked at whether, contrariwise, effects could be found in patients who shifted from REM to LDA (*n* = 22), HAQ indeed deteriorated significantly (REM, 0.4 ± 0.4; LDA, 0.5 ± 0.4; *P* = 0.014). We found no change in HRQoL or productivity in this situation (results not shown).

## Discussion

In this study, we observed significant differences in functional disability, health-related quality of life, fatigue, and work productivity between different levels of disease activity, showing an increase of functional impairment and fatigue and a decrease of work productivity and health-related quality of life with worsening disease-activity states. Although it was not surprising to find major differences between higher and lower states of disease activity, the major finding of our study relates to the significant differences between LDA and REM across all variables assessed: physical function, health-related quality of life, work capacity, and costs. Moreover, these findings from cross-sectional analyses were also supported in a small group of patients that allowed the longitudinal changes when moving from LDA to REM and vice versa.

Recently, new treatment recommendations have been published, taking into account economic aspects of treatment options in RA [12]. A systematic literature review published in 2010 [14] indicated that treatment strategies leading to maintenance of physical function and keeping patients at work are cost effective, even when including more-costly biologic agents. Even though leading to a significant increase of medical costs [33], biologic agents can substantially improve disease activity and reduce progression of joint destruction [34-41], especially in patients at high risk to develop these.

Treating rheumatoid arthritis to the target of remission has been proposed to constitute an optimal therapeutic goal [12]. However, whereas low disease activity constitutes an alternative goal, especially in patients with longstanding disease, only insufficient data exist on whether the socioeconomic implications of low disease activity are similar to or different from those of remission. Answering this question will ultimately be the driver of the decision whether it is worthwhile aiming for remission in patients who already have reached an acceptable clinical state: low disease activity. In our study, we showed that in all aspects of patient-reported outcomes, remission is superior to low disease activity, regardless of the index used. Furthermore, we observed that even in patients who already had reached low disease activity, an improvement of these outcomes was seen upon reaching remission.

It is noteworthy that patients in REM on average did not reach normal health-related quality of life values, which contrasts with other findings [42]. This is presumably due to the long disease duration of our patients (mean of about 11 years) and thus the accrual of irreversible damage and disability [8]. However, this might also make the findings even more significant.

Several limitations of our study should be addressed. First, the evaluation of indirect costs was estimated by using previously published cost analyses rather than self-evaluation of sick-leave and work-disability costs. Although several studies have revealed that functional disability is highly correlated with and a major predictor of indirect costs, these estimated costs should be regarded as a guidance value rather than an individualized cost calculation. Conversely, by using the WPAI, we could substantiate our findings, showing an increase of “degree RA affected productivity while working” with increasing level of disease activity. Because of recall bias, we did not ask patients to report the whole previous year but only 3 months, and to explore costs for a whole year by multiplying it, which is only an approximation of the actual costs and resource utilizations. The fact that only about 26% of our population was currently employed might overestimate the findings on productivity, although the percentage reflects a typical RA cohort.

We must address that we found a significantly lower percentage of overall work impairment due to RA when compared with results of other studies [25,43]. Therefore, the results on work impairment must be interpreted with caution and might not be generalizable and assignable to other cohorts.

Second, when assessing direct medical costs, we reported only costs due to synthetic or biologic DMARDs but disregarded costs due to other medications, such as glucocorticoids, NSAIDs, or drugs used to treat RA-related comorbidities.

Third, we studied only relatively few patients longitudinally; however, despite this small number, the differences between LDA and REM were statistically significant in both directions (improvement from LDA to REM and deterioration from REM to LDA) and thus support the cross-sectional analyses. Still, further research is needed, to strengthen and validate our longitudinal findings, that going from low disease activity to remission leads to significant improvement of important outcomes like physical function, but also HRQoL or productivity. The finding that when using DAS28, HAQ levels in REM and LDA did not differ significantly, does not decrease the validity of the results. Because DAS28 remission is not sufficiently stringent [44], and given the low range of values for classification of a DAS28 LDA state, the similarity of results between REM and LDA when using DAS28 is not surprising.

Thus, our focus on the more-stringent SDAI remission, which constitutes the ACR-EULAR index-based remission definition [44], allowed recognition of the differences between REM and all other states, including LDA. Also, the fact that our study relates to the inclusion of a large sample of clinic patients with a broad range of disease activity, disease duration, treatment strategies, and comorbidities, rather than more homogeneous trial populations, allows insights into the routine care of RA patients.

Of additional importance, our analyses revealed that LDA is a much better state than MDA/HDA in all respects: physical function, health-related quality of life, and productivity. Thus, indeed, LDA is a good alternative option for patients in whom remission cannot be reached either due to their long-standing, refractory disease or to the inability to intensify therapy due to comorbidity, contraindications, or patient preference. Therefore, although the present data unequivocally support the value of reaching remission and should be used in counseling patients and rheumatologists on the importance of assessing disease activity and targeting stringent remission where this goal is achievable, they should not be used to justify intensification of treatment where only a little is to be gained or risks prevail.

## Conclusion

Reaching remission seems to be a desirable state from patients’ as well as socioeconomic perspectives. Patients in remission appear to have higher health-related quality of life, better physical function, and greater work capacity compared with those with low disease activity. From an economic perspective, the direct as well as the indirect costs are lower in patients attaining remission compared with those in higher states of disease activity, even including low disease activity. Therefore, aiming for remission remains the ultimate goal when treating RA patients, but a decision for which efforts will be used to move patients from low disease activity to remission will require careful considerations, based not only on patient preferences and contraindications, but also on cost thresholds that society will have to define.

## Abbreviations

ACR: American College of Rheumatology; ANOVA: analysis of variance; BP: bodily pain; CDAI: Clinical Disease Activity Index; CRP: C-reactive protein; DAS28: Disease Activity Score using 28-joint count; DMARD: disease-modifying antirheumatic drug; EGA: Evaluator Global Assessment of Disease Activity; EMM: estimated marginal means; EQ-5D: Euro-QoL 5 dimensions; ESR: erythrocyte sedimentation rate; EULAR: European League Against Rheumatism; FCA: friction cost approach; GHP: general health perception; GLM: general linear model; HAQ: health-assessment questionnaire; HCA: human capital approach; HDA: high disease activity; HRQoL: health-related quality of life; LDA: low disease activity; MCS: Mental Component Score; MDA: moderate disease activity; MH: mental health; NSAID: nonsteroidal antiinflammatory drug; PCS: Physical Component Score; PF: physical function; PGA: Patient Global Assessment of Disease Activity; r: Spearman correlation coefficient; RA: rheumatoid arthritis; RE: Role Emotional; REM: remission; RP: Role Physical; SD: standard deviation; SDAI: Simplified Disease Activity Index; SF: Social Function; SF-36: Short Form 36; SF-6D: Short Form 6 dimensions; SJC28: Swollen Joint Count 28; TJC28: Tender Joint Count 28; VAS: visual analogue scale; VT: vitality; WPAI: Work Productivity and Activity Impairment Questionnaire.

## Competing interests

The study was supported in part by a grant from UCB (Belgium). The authors declare that they have no competing interests.

## Authors’ contributions

HR provided conception and design, data collection, performance of all statistical analyses, manuscript writing, and final approval of the manuscript. JS participated in conception and design, manuscript writing, and final approval of the manuscript. DA provided conception and design, manuscript writing, and final approval of the manuscript. All authors reviewed the manuscript before submission and approved the final version.

## Supplementary Material

Additional file 1 **Questionnaire used in the study to assess indirect and direct costs.** The items collected were based on cost-effectiveness article and a systematic literature review on cost in RA. Click here for file

## References

[B1] Kobelt G Jonsson B The burden of rheumatoid arthritis and access to treatment: outcome and cost-utility of treatments Eur J Health Econ 2008 16 95 106 10.1007/s10198-007-0091-0 18157559

[B2] Pugner KM Scott DI Holmes JW Hieke K The costs of rheumatoid arthritis: an international long-term view Semin Arthritis Rheum 2000 16 305 320 10.1016/S0049-0172(00)80017-7 10805355

[B3] Fries JF Safety, cost and effectiveness issues with disease modifying anti-rheumatic drugs in rheumatoid arthritis Ann Rheum Dis 1999 16 I86 I89 10.1136/ard.58.2008.i86 10577980 PMC1766585

[B4] Smolen JS Aletaha D Koeller M Weisman MH Emery P New therapies for treatment of rheumatoid arthritis Lancet 2007 16 1861 1874 10.1016/S0140-6736(07)60784-3 17570481

[B5] Kvamme MK Lie E Kvien TK Kristiansen IS Two-year direct and indirect costs for patients with inflammatory rheumatic joint diseases: data from real-life follow-up of patients in the NOR-DMARD registry Rheumatology (Oxford) 2012 16 1618 1627 10.1093/rheumatology/kes074 22539479

[B6] Aletaha D Funovits J Breedveld FC Sharp J Segurado O Smolen JS Rheumatoid arthritis joint progression in sustained remission is determined by disease activity levels preceding the period of radiographic assessment Arthritis Rheum 2009 16 1242 1249 10.1002/art.24433 19404938

[B7] Smolen JS Han C van der Heijde DM Emery P Bathon JM Keystone E Maini RN Kalden JR Aletaha D Baker D Han J Bala M St Clair EW Radiographic changes in rheumatoid arthritis patients attaining different disease activity states with methotrexate monotherapy and infliximab plus methotrexate: the impacts of remission and tumour necrosis factor blockade Ann Rheum Dis 2009 16 823 827 10.1136/ard.2008.090019 18593759

[B8] Aletaha D Smolen J Ward MM Measuring function in rheumatoid arthritis: identifying reversible and irreversible components Arthritis Rheum 2006 16 2784 2792 10.1002/art.22052 16947781

[B9] Kavanaugh A Han C Bala M Functional status and radiographic joint damage are associated with health economic outcomes in patients with rheumatoid arthritis J Rheumatol 2004 16 849 855 15124242

[B10] Smolen JS Han C van der Heijde D Emery P Bathon JM Keystone E Kalden JR Schiff M Bala M Baker D Infliximab treatment maintains employability in patients with early rheumatoid arthritis Arthritis Rheum 2006 16 716 722 10.1002/art.21661 16508932

[B11] Combe B Landewe R Lukas C Bolosiu HD Breedveld F Dougados M Emery P Ferraccioli G Hazes JM Klareskog L EULAR recommendations for the management of early arthritis: report of a task force of the European Standing Committee for International Clinical Studies Including Therapeutics (ESCISIT) Ann Rheum Dis 2007 16 34 45 16396980 10.1136/ard.2005.044354 PMC1798412

[B12] Smolen JS Aletaha D Bijlsma JW Breedveld FC Boumpas D Burmester G Combe B Cutolo M de Wit M Dougados M Treating rheumatoid arthritis to target: recommendations of an international task force Ann Rheum Dis 2010 16 631 637 10.1136/ard.2009.123919 20215140 PMC3015099

[B13] Lundkvist J Kastang F Kobelt G The burden of rheumatoid arthritis and access to treatment: health burden and costs Eur J Health Econ 2008 16 S49 S60 10.1007/s10198-007-0088-8 18157732

[B14] Schoels M Wong J Scott DL Zink A Richards P Landewe R Smolen JS Aletaha D Economic aspects of treatment options in rheumatoid arthritis: a systematic literature review informing the EULAR recommendations for the management of rheumatoid arthritis Ann Rheum Dis 2010 16 995 1003 10.1136/ard.2009.126714 20447950

[B15] Stamm TA Aletaha D Pflugbeil S Kapral T Montag K Machold KP Smolen JS The use of databases for quality assessment in rheumatoid arthritis Clin Exp Rheumatol 2007 16 82 85 18021511

[B16] Aletaha D Nell VP Stamm T Uffmann M Pflugbeil S Machold K Smolen JS Acute phase reactants add little to composite disease activity indices for rheumatoid arthritis: validation of a clinical activity score Arthritis Res Ther 2005 16 R796 R806 10.1186/ar1740 15987481 PMC1175030

[B17] Smolen JS Breedveld FC Schiff MH Kalden JR Emery P Eberl G van Riel PL Tugwell P A simplified disease activity index for rheumatoid arthritis for use in clinical practice Rheumatology (Oxford) 2003 16 244 257 10.1093/rheumatology/keg072 12595618

[B18] Prevoo ML Hof MAVt Kuper HH van Leeuwen MA van de Putte LB van Riel PL Modified disease activity scores that include twenty-eight-joint counts: development and validation in a prospective longitudinal study of patients with rheumatoid arthritis Arthritis Rheum 1995 16 44 48 10.1002/art.1780380107 7818570

[B19] Aletaha D Ward MM Machold KP Nell VP Stamm T Smolen JS Remission and active disease in rheumatoid arthritis: defining criteria for disease activity states Arthritis Rheum 2005 16 2625 2636 10.1002/art.21235 16142705

[B20] Huscher D Merkesdal S Thiele K Zeidler H Schneider M Zink A Cost of illness in rheumatoid arthritis, ankylosing spondylitis, psoriatic arthritis and systemic lupus erythematosus in Germany Ann Rheum Dis 2006 16 1175 1183 10.1136/ard.2005.046367 16540552 PMC1798296

[B21] McHorney CA Ware JE Jr Raczek AE The MOS 36-Item Short-Form Health Survey (SF-36): II, psychometric and clinical tests of validity in measuring physical and mental health constructs Med Care 1993 16 247 263 10.1097/00005650-199303000-00006 8450681

[B22] Ware JE Jr Sherbourne CD The MOS 36-item short-form health survey (SF-36): I. conceptual framework and item selection Med Care 1992 16 473 483 10.1097/00005650-199206000-00002 1593914

[B23] Brazier J Usherwood T Harper R Thomas K Deriving a preference-based single index from the UK SF-36 Health Survey J Clin Epidemiol 1998 16 1115 1128 10.1016/S0895-4356(98)00103-6 9817129

[B24] The EuroQol Group EuroQoL: a new facility for the measurement of health-related quality of life Health Policy 1990 16 199 208 10109801 10.1016/0168-8510(90)90421-9

[B25] Zhang W Bansback N Boonen A Young A Singh A Anis AH Validity of the work productivity and activity impairment questionnaire: general health version in patients with rheumatoid arthritis Arthritis Res Ther 2010 16 R177 10.1186/ar3141 20860837 PMC2991008

[B26] Li LC Maetzel A Davis AM Lineker SC Bombardier C Coyte PC Primary therapist model for patients referred for rheumatoid arthritis rehabilitation: a cost-effectiveness analysis Arthritis Rheum 2006 16 402 410 10.1002/art.21989 16739183

[B27] Rosery H Bergemann R Maxion-Bergemann S International variation in resource utilisation and treatment costs for rheumatoid arthritis: a systematic literature review Pharmacoeconomics 2005 16 243 257 10.2165/00019053-200523030-00005 15836006

[B28] Johannesson M The willingness to pay for health changes, the human-capital approach and the external costs Health Policy 1996 16 231 244 10.1016/0168-8510(96)00815-9 10158268

[B29] Brouwer WB Koopmanschap MA The friction-cost method: replacement for nothing and leisure for free? Pharmacoeconomics 2005 16 105 111 10.2165/00019053-200523020-00002 15748085

[B30] Koopmanschap MA Rutten FF van Ineveld BM van Roijen L The friction cost method for measuring indirect costs of disease J Health Econ 1995 16 171 189 10.1016/0167-6296(94)00044-5 10154656

[B31] Aletaha D Smolen JS Joint damage in rheumatoid arthritis progresses in remission according to the Disease Activity Score in 28 joints and is driven by residual swollen joints Arthritis Rheum 2011 16 3702 3711 10.1002/art.30634 21953215

[B32] Felson DT Smolen JS Wells G Zhang B van Tuyl LH Funovits J Aletaha D Allaart CF Bathon J Bombardieri S American College of Rheumatology/European League Against Rheumatism provisional definition of remission in rheumatoid arthritis for clinical trials Arthritis Rheum 2011 16 573 586 10.1002/art.30129 21294106 PMC3115717

[B33] Michaud K Messer J Choi HK Wolfe F Direct medical costs and their predictors in patients with rheumatoid arthritis: a three-year study of 7,527 patients Arthritis Rheum 2003 16 2750 2762 10.1002/art.11439 14558079

[B34] Breedveld FC Weisman MH Kavanaugh AF Cohen SB Pavelka K van Vollenhoven R Sharp J Perez JL Spencer-Green GT The PREMIER study: a multicenter, randomized, double-blind clinical trial of combination therapy with adalimumab plus methotrexate versus methotrexate alone or adalimumab alone in patients with early, aggressive rheumatoid arthritis who had not had previous methotrexate treatment Arthritis Rheum 2006 16 26 37 10.1002/art.21519 16385520

[B35] Keystone E Heijde D Mason D Jr Landewe R Vollenhoven RV Combe B Emery P Strand V Mease P Desai C Certolizumab pegol plus methotrexate is significantly more effective than placebo plus methotrexate in active rheumatoid arthritis: findings of a fifty-two-week, phase III, multicenter, randomized, double-blind, placebo-controlled, parallel-group study Arthritis Rheum 2008 16 3319 3329 10.1002/art.23964 18975346

[B36] Klareskog L van der Heijde D de Jager JP Gough A Kalden J Malaise M Martin Mola E Pavelka K Sany J Settas L Therapeutic effect of the combination of etanercept and methotrexate compared with each treatment alone in patients with rheumatoid arthritis: double-blind randomised controlled trial Lancet 2004 16 675 681 10.1016/S0140-6736(04)15640-7 15001324

[B37] Kremer JM Westhovens R Leon M Di Giorgio E Alten R Steinfeld S Russell A Dougados M Emery P Nuamah IF Treatment of rheumatoid arthritis by selective inhibition of T-cell activation with fusion protein CTLA4Ig N Engl J Med 2003 16 1907 1915 10.1056/NEJMoa035075 14614165

[B38] Lipsky PE van der Heijde DM St Clair EW Furst DE Breedveld FC Kalden JR Smolen JS Weisman M Emery P Feldmann M Infliximab and methotrexate in the treatment of rheumatoid arthritis: Anti-Tumor Necrosis Factor Trial in Rheumatoid Arthritis with Concomitant Therapy Study Group N Engl J Med 2000 16 1594 1602 10.1056/NEJM200011303432202 11096166

[B39] Smolen JS Beaulieu A Rubbert-Roth A Ramos-Remus C Rovensky J Alecock E Woodworth T Alten R Effect of interleukin-6 receptor inhibition with tocilizumab in patients with rheumatoid arthritis (OPTION study): a double-blind, placebo-controlled, randomised trial Lancet 2008 16 987 997 10.1016/S0140-6736(08)60453-5 18358926

[B40] Smolen JS Han C Bala M Maini RN Kalden JR van der Heijde D Breedveld FC Furst DE Lipsky PE Evidence of radiographic benefit of treatment with infliximab plus methotrexate in rheumatoid arthritis patients who had no clinical improvement: a detailed subanalysis of data from the anti-tumor necrosis factor trial in rheumatoid arthritis with concomitant therapy study Arthritis Rheum 2005 16 1020 1030 10.1002/art.20982 15818697

[B41] van der Heijde D Kalden J Scott D Smolen J Strand V Long term evaluation of radiographic disease progression in a subset of patients with rheumatoid arthritis treated with leflunomide beyond 2 years Ann Rheum Dis 2004 16 737 739 10.1136/ard.2003.010983 15140783 PMC1755041

[B42] Linde L Sorensen J Ostergaard M Horslev-Petersen K Hetland ML Does clinical remission lead to normalization of EQ-5D in patients with rheumatoid arthritis and is selection of remission criteria important? J Rheumatol 2010 16 285 290 10.3899/jrheum.090898 20080905

[B43] Chaparro Del Moral R Rillo OL Casalla L Moron CB Citera G Cocco JA Correa Mde L Buschiazzo E Tamborenea N Mysler E Tate G Banos A Herscovich N Work productivity in rheumatoid arthritis: relationship with clinical and radiological features Arthritis 2012 16 137635 [published online] 23320166 10.1155/2012/137635 PMC3535832

[B44] Felson DT Smolen JS Wells G Zhang B van Tuyl LH Funovits J Aletaha D Allaart CF Bathon J Bombardieri S Brooks P Brown A Matucci-Cerinic M Choi H Combe B de Wit M Dougados M Emery P Furst D Gomez-Reino J Hawker G Keystone E Khanna D Kirwan J Kvien TK Landewe R Listing J Michaud K Martin-Mola E Montie P American College of Rheumatology/European League Against Rheumatism provisional definition of remission in rheumatoid arthritis for clinical trials Ann Rheum Dis 2011 16 404 413 10.1136/ard.2011.149765 21292833

