# Supplementary material for: Remission in rheumatoid arthritis: benefit over low disease activity in patient-reported outcomes and costs
Source: Arthritis Res Ther. 2014 Feb 21;16(1):R56. doi: 10.1186/ar4491 (PMC3979137; doi:10.1186/ar4491)
Supplement: Additional file 1 — Questionnaire used in the study to assess indirect and direct costs. The items collected were based on cost-effectiveness article and a systematic literature review on cost in RA. [file ar4491-S1.pdf]

Die nächsten Fragen beziehen sich auf den ***Zeitraum zwischen der letzten regulären und der heutigen Visite*** *in unserer Rheumaambulanz*

17. STATIONÄRE KRANKENHAUSAUFENTHALTE (mindestens 1 Nacht)

Wurden Sie seit Ihrer letzten Visite *aufgrund Ihrer chronischen Polyarthritis* stationär in einem Krankenhaus behandelt?

☐ JA ☐ NEIN

Wenn JA, wie häufig: ____mal

wie viele Tage insgesamt: _____Tage

Grund des Aufenthalts: ☐ Schub der chronischen Polyarthritis

☐ Gelenksoperation

☐ Medikamentenunverträglichkeit

☐ Sonstige______________________

18. TAGESKLINIK (Ohne Nächtigung im Krankenhaus)

Wurden Sie seit Ihrer letzten Visite *aufgrund Ihrer chronischen Polyarthritis* stationär behandelt ohne im Krankenhaus zu übernachten („Tagesstation")?

☐ JA ☐ NEIN

Wenn JA:

Wie häufig: ______mal

Aus welchem Grund? ____

19. UNTERSUCHUNGEN

Wurden bei Ihnen *aufgrund Ihrer chronischen Polyarthritis* seit der letzten Visite spezielle Untersuchungen durchgeführt?

☐ JA ☐ NEIN

Wenn JA, kreuzen Sie bitte an welche (Mehrfachnennung möglich):

☐ Röntgen	☐ Arthroskopie
☐ Computertomographie CT	☐ Ultraschalluntersuchung Gelenke
☐ Kernspintomographie MRT	☐ Ultraschalluntersuchung andere ____________
☐ Knochendichtemessung	☐ Sonstiges _____________

20. REHABILITATION / KURAUFENTHALTE

Hatten Sie *aufgrund Ihrer chronischen Polyarthritis* seit Ihrer letzten Visite Rehabilitations- oder Kuraufenthalte?

☐ JA ☐ NEIN

Wenn JA, wie viele Tage insgesamt: ______Tage

wie viele Kosten fielen für Sie an (Selbstbehalt) _____________ €

21. AMBULUANTE BEHANDLUNG (Krankenhausambulanzen)

Wurden Sie seit Ihrer letzten Visite *aufgrund Ihrer chronischen Polyarthritis* ambulant in einem Krankenhaus behandelt?

☐ JA ☐ NEIN

Wenn JA, kreuzen Sie bitte an, in welcher Ambulanz Sie waren (Mehrfachnennung möglich) und geben Sie bitte an wie oft:

- ☐ Rheumaambulanz ____ mal
- ☐ Orthopädische Ambulanz ____ mal
- ☐ Augenambulanz ____mal
- ☐ Hautambulanz ____mal
- ☐ Gefäßambulanz ____mal
- ☐ Sonstiges __________________ ____mal

22. ARZTBESUCHE (niedergelassener Bereich)

Waren Sie seit Ihrer letzten Visite *aufgrund Ihrer chronischen Polyarthritis* in der Ordination eines Arztes in Behandlung?

☐ JA ☐ NEIN

Wenn JA, kreuzen Sie bitte an, bei wem Sie waren (Mehrfachnennung möglich) und geben Sie bitte an wie oft:

- ☐ Praktischer Arzt ____mal
- ☐ Rheumatologe ____mal
- ☐ Orthopäde ____mal
- ☐ Augenarzt ____mal
- ☐ Internist ____mal
- ☐ sonstige______________ ___mal

23. WEITERE SPEZIALISTEN

Wurden seit Ihrer letzten Visite *aufgrund Ihrer chronischen Polyarthritis* nicht-medikamentöse oder spezielle Behandlungen (z.B. Physiotherapie) durchgeführt?

☐ JA ☐ NEIN

Wenn JA, kreuzen Sie bitte an, welche durchgeführt wurden (Mehrfachnennung möglich) und geben Sie bitte an wie oft diese durchgeführt wurden. Geben Sie bitte auch durch Ankreuzen an ob Ihre Krankenkasse (KK) dafür aufkam, bzw. nennen Sie uns bitte den ungefähren Betrag, den Sie selbst bezahlen mussten:

☐ Ergotherapie ____mal

☐ KK bezahlt ☐ Selbst bezahlt €_________

☐ Physiotherapie (z.B. Heilgymnastik) ____mal

☐ KK bezahlt ☐ Selbst bezahlt €_________

☐ Physikalische Therapie (z.B. Strom-, Ultraschalltherapie) ___mal

☐ KK bezahlt ☐ Selbst bezahlt €_________

☐ Akupunktur ____mal

☐ KK bezahlt ☐ Selbst bezahlt €_________

☐ Chiropraktiker ____mal

☐ KK bezahlt ☐ Selbst bezahlt €_________

☐ Massagen ____mal

☐ KK bezahlt ☐ Selbst bezahlt €_________

☐ Psychotherapie ___mal

☐ KK bezahlt ☐ Selbst bezahlt €_________

☐ Homöopath ___ mal

☐ KK bezahlt ☐ Selbst bezahlt €_________

☐ Sonstige____________________ ___mal

☐ KK bezahlt ☐ Selbst bezahlt €________

24. BLUTUNTERSUCHUNGEN

Wurden *aufgrund Ihrer chronischen Polyarthritis* seit Ihrer letzten Visite Blutuntersuchungen / Laborkontrollen abseits der oben genannten Ambulanz- oder Arztbesuche durchgeführt?

☐ JA ☐ NEIN

Wenn JA, geben Sie bitte an wie oft: _____ mal

25. GELENKOPERATIONEN

Wurden *aufgrund Ihrer chronischen Polyarthritis* seit Ihrer letzten Visite Operationen an Gelenken durchgeführt?

☐ JA ☐ NEIN

Wenn JA, geben Sie bitte an welche(s) Gelenk(e) ______________________________

26. BAULICHE VERÄNDERUNGEN UND HILFSMITTEL

Sind seit Ihrer letzten Visite *aufgrund Ihrer chronischen Polyarthritis* bauliche oder technische Veränderungen in Ihrer häuslichen Umgebung notwendig gewesen oder mussten Sie sich andere technische Hilfsmittel zulegen?

☐ JA ☐ NEIN

Wenn JA, kreuzen Sie bitte an welche (Mehrfachnennung möglich):

- ☐ Bauliche Veränderungen Haus / Wohnung (Rampen, Lift,…)
- ☐ Badezimmerveränderungen wie Duschsitz, Toilettensitzerhöhung, Haltegriffe
- ☐ Technische Veränderungen am Auto
- ☐ Schienen für Hände
- ☐ Orthopädische Maßnahmen für Füße wie Schuheinlagen, Absatzerhöhung, ..
- ☐ Gehhilfen wie Stöcke, Krücken,…
- ☐ Arbeitshilfen
- ☐ Hilfsmittel zur Alltagsbewältigung wie Besteck, Dosenöffner, Griffverdickungen, Greifhilfe,..
- ☐ Sonstiges ____________________

Wie viel Geld haben Sie diese Maßnahmen zusammen ungefähr gekostet?

____________€ ☐ Krankenkasse bezahlt ☐ weiß nicht

27. TRANSPORTKOSTEN

Fielen seit Ihrer letzten Visite *aufgrund der chronischen Polyarthritis* Reise- bzw. Transportkosten an (z. B Anreise zu Arztbesuchen, Ambulanzvisiten, Therapiestunden, etc.)?

☐ JA ☐ NEIN ☐ Anreise mit Krankentransport (Rettung)

Wenn JA:

a. Wenn das Auto verwendet wurde, bitte schätzen Sie, wie viele Kilometer Sie insgesamt dafür aufbrachten.

________km ☐ weiß nicht

b. Wenn Sie Taxi, Bus, Zug oder sonstiges verwendet haben, bitte schätzen Sie, wie viele Kosten für Sie insgesamt anfielen.

€ _________ ☐ weiß nicht

28. HEIMHILFE

Benötigten Sie seit Ihrer letzten Visite *aufgrund der chronischen Polyarthritis* irgendwelche Art von Pflege oder Heimhilfe?

☐ JA ☐ NEIN

Wenn JA, kreuzen Sie bitte an welche Art von Pflege / Heimhilfe benötigt wurde (Mehrfachnennung möglich):

☐ Pflege von Familienangehörigen / Freunden

☐ Pflege durch professionelle Heimhilfen / mobile Krankenpflege

☐ Putzfrau, Haushaltshilfe

☐ andere___________________

a. Wenn Sie Pflege oder Hilfe von Familienangehörigen / Freunden oder anderen erhalten haben, wie viele Stunden pro Woche wurde dafür aufgewendet?

______ h/Woche ☐ weiß nicht

b. Wenn Sie Pflege / Hilfe durch eine professionelle Heimhilfe, mobile Krankenpflege oder Putzfrau bekommen, wie viel Kosten pro Woche fallen dadurch für Sie an?

______ €/Woche ☐ weiß nicht
